# Antimicrobial nanocomposite adsorbent based on poly(meta-phenylenediamine) for remediation of lead (II) from water medium

**DOI:** 10.1038/s41598-022-08668-1

**Published:** 2022-03-17

**Authors:** Fatemeh Bandavi Kheyrabadi, Ehsan Nazarzadeh Zare

**Affiliations:** School of Chemistry, Damghan University, P.O. Box, 36716-41167 Damghan, Iran

**Keywords:** Environmental chemistry, Chemistry, Environmental chemistry

## Abstract

In this study, poly(m-phenylenediamine)@ZnO (PmPDA@ZnO) nanocomposite was fabricated by in-situ chemical oxidative polymerization for the effective lead(II) removal from aqueous solutions. PmPDA@ZnO was characterized by several instrumental methods like FTIR, XRD, EDX, TGA, FESEM, TEM, zeta potential, and BET. The TEM images showed a core–shell-like structure for the PmPDA@ZnO nanocomposite. TGA results showed that the thermal stability of the PmPDA@ZnO nanocomposite was higher than the PmPDA. The maximum adsorption of lead (II) onto PmPDA@ZnO nanocomposite was obtained at pH 6, adsorbent dosage 60 mg, lead(II) ion concentration 90 mg/L, and agitation time 90 min. Langmuir and Freundlich's isotherm models were evaluated to simulate the lead(II) sorption via empirical data. Langmuir's model was in good agreement with empirical data with a maximum adsorption capacity (Q_max_) of 77.51 mg/g. The kinetic data adsorption fitted best the pseudo-second-order model. The values of thermodynamic parameters of ΔS° and ΔH° were obtained 0.272 J/mol K, and 71.35 kJ/mol, respectively. The spontaneous and endothermic behavior of the adsorption process was confirmed by the negative and positive response of ΔG° and ΔH°, respectively. Moreover, the addition of coexisting cations e.g. cobalt (II), nickel (II), calcium (II), and copper (II) had no significant effect on the removal efficiency of lead(II). Adsorption–desorption studies showed that the PmPDA@ZnO nanocomposite can be remarkably regenerated and reused after three sequential runs without a significant decline in its adsorption performance. The antimicrobial activities of PmPDA@ZnO nanocomposite were evaluated against *Escherichia coli* and *Staphylococcus aureus* bacteria species. These results confirmed that the PmPDA@ZnO nanocomposite could be a good candidate for water decontamination.

## Introduction

Nowadays, water shortage is one of the important problems in humane societies. Only 0.041% of all of the earth's water is fresh and is accessible^1^. About 97% of the remaining water is saline, and access to less than 3% of water is difficult^2^. In addition, about 22% of the world population, possesses only 7% of global freshwater resources. A geographic and temporal mismatch between demand and access to freshwater are major causes of global water scarcity. On the other hand, water pollution is one of the most important management challenges in the world^3^. Population growth and industrial development led to increased pollution of surface and groundwater^4^. The existence of pollutants such as drugs (hospital wastewater), metal ions (metallurgical industry), dyes (textile industry), agricultural pesticides, etc. led to contamination of aquatic ecosystems and their adverse impacts^5–10^. Contaminant accumulation in human organs causes serious diseases like bladder cancer, testicle cancer, uterus cancer, blood cancer, as well as diseases such as anemia, etc. Lead is one of the toxic heavy metals that accumulation in the human body causes severe damage to the kidney, nervous system, reproductive system, liver, and brain. Lead is generally produced from pesticides, mobile batteries, and petroleum-based materials. The allowable level for lead in drinking water is 0.05 mg/L^11,12^. Consequently, removing lead from contaminated water is essential. Till now, numerous methods e.g., ion exchange, adsorption, chemical precipitation, coagulation–sedimentation, reverse osmosis, etc. have been developed to remove heavy metals from contaminated water. Among them, adsorption is widely employed owing to its low method cost, high removal effectiveness, and ease of process^13^. Many natural (e.g. agricultural residues, activated carbon, natural polymers) and synthetic adsorbents (e.g. polymers and mineral nanoparticles) have been employed for the treatment of contaminated water^14–16^. Polymer-based nanocomposite sorbents have been displayed an effective role in water purification. These materials showed better chemical-physical properties than one-component-based adsorbents; for instance, they display improved mechanical, thermal, and optical properties, as well as have a broader range of selectivity for the removal of the contamination^17,18^. Among polymer-based nanocomposites, conducting polymer-based adsorbents, such as polyaniline and its derivatives (e.g. ortho-, meta-, and para-phenylenediamines) have received important attention owing to their potential applications in adsorbing different heavy metal ions, ease of synthesis, porous structure, regeneration, non-toxicity, environmental and mechanical stability, and low cost^19,20^. Poly(phenylenediamine)s (such as ortho-, meta-, and para) have received more attention in water treatment due to the presence of two amine complexing agents^19,21–26^. On the other hand, nanomaterials such as iron oxide, silver, zinc oxide, copper oxide, etc. are the most usually used for the adsorption of toxic metals ion^27–30^. Zinc oxide has exposed great performance for removal of metal ions, generally in simultaneous removal of inorganic and organic pollutants and as an antimicrobial agent^31^. On the other side, dangerous microbes were also lead to cause numerous health infections in humans. Therefore, the preparation of antimicrobial nanocomposite adsorbents for water decontamination is of considerable interest^32^.

**Scheme 1:**
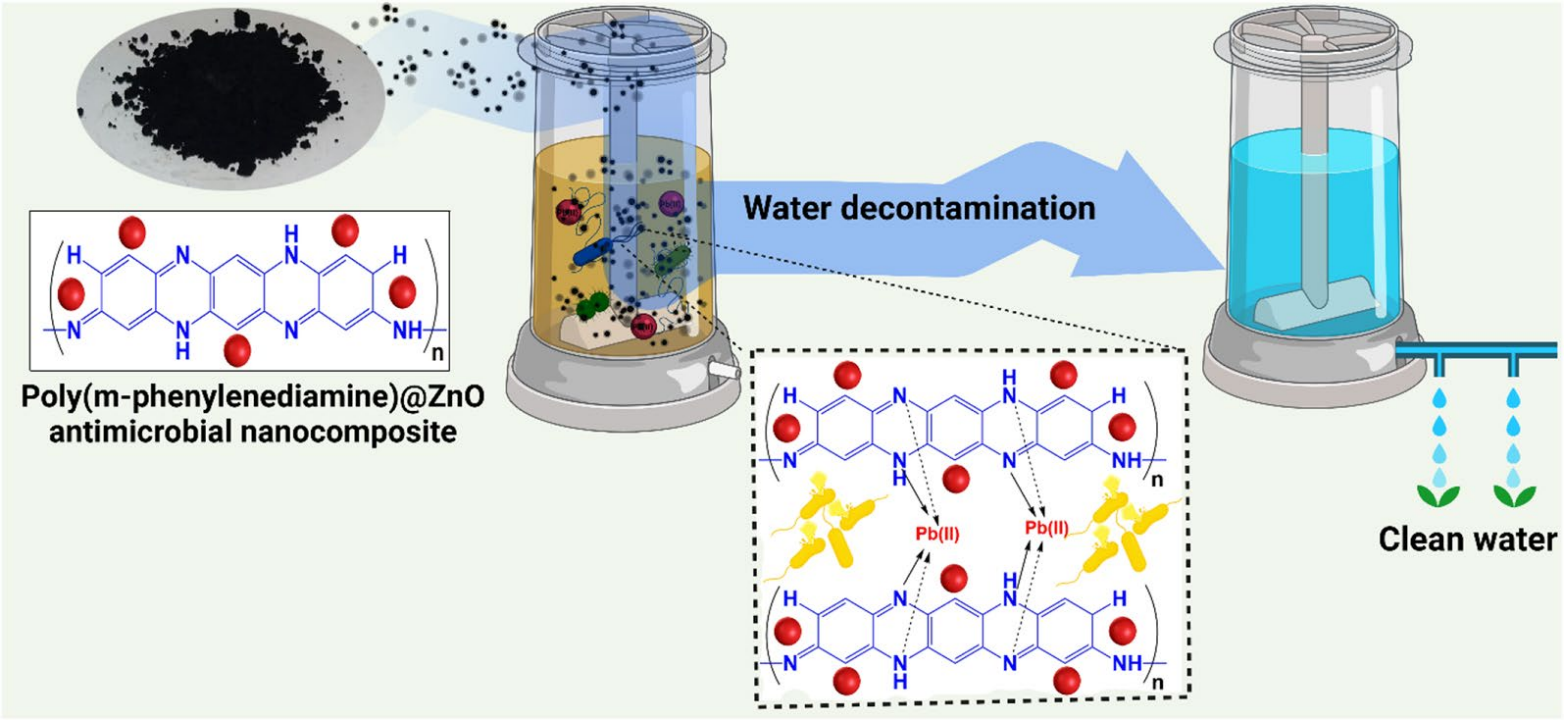
Schematic demonstration of antimicrobial poly(meta-phenylenediamine)@ZnO nanocomposite for water decontamination.

In the current work, an organic/inorganic antimicrobial nanocomposite based on poly(meta-phenylenediamine) and ZnO nanoparticles was fabricated via in-situ chemical oxidative polymerization for the removal of lead(II) from contaminated water (Scheme 1). The influences parameters on adsorption such as agitation time, solution pH, lead(II) initial concentration, and adsorbent dosage on the sorption process were evaluated. Isotherm, kinetics, and thermodynamics models were also studied. The antimicrobial activity of ZnO nanoparticles and poly(meta-phenylenediamine)@ZnO nanocomposite was also evaluated against *Escherichia coli* (a Gram-negative) and *Staphylococcus aureus* (a Gram-positive).

## Materials and methods

### Materials

Meta-phenylenediamine (mPDA), ammonium persulfate (APS), hydrochloric acid, ammonia solution, and all used solvents were provided from Merck Company (Germany). Spherical zinc oxide nanoparticles (ZnO NPs) with a mean diameter of ~ 40 nm were provided from Neutrino Company (Tehran, Iran).

### Fabrication of poly(m-phenylenediamine)@ZnO nanocomposite

The poly(m-phenylenediamine)@ZnO (PmPDA@ZnO) nanocomposite was fabricated by in-situ chemical oxidative polymerization at optimal conditions as follows: In a round-bottomed flask, 1 g of mPDA was dissolved in 30 mL of hydrochloric acid solution (0.1 M). Afterward, the solution was degassed by nitrogen for 15 min. Subsequently, 0.1 g (10 wt% to the mPDA) of ZnO NPs in 5 mL of distilled water were sonicated until completely dispersed and then were added to the above solution. A solution of APS (2.072 g in 15 mL of hydrochloric acid solution) was added to the above solution drop by drop for 15 min. The final solution was kept under magnetic stirring for 24 h to obtain PmPDA@ZnO nanocomposite. The resulting precipitate was washed with water and acetone and air-dried. For the preparation of undoped PmPDA@ZnO nanocomposite (deprotonation of the PmPDA@ZnO nanocomposite), the precipitate was added to the ammonia solution (100 mL, 2 M) under magnetic stirring at room temperature for 4 h, then the precipitate was washed several times with water and methanol. For better comparison, PmPDA was also synthesized by the above reaction and then undoped by ammonia solution (2 M).

### Characterization

Fourier transform infrared spectroscopy (FTIR) (Equinox 55, Bruker Optik GmbH), and energy-dispersive X-ray (EDX) analysis (MIRA 3-XMU, Tescan, Kohoutovice) were employed for approval of the chemical structure of polymer and nanocomposite. X-ray diffraction (XRD) (D8 Advance X-ray diffractometer, Bruker Optik GmbH), field emission scanning electron microscope (FESEM) (MIRA 3-XMU, Tescan, Kohoutovice), and transmission electron microscope (Philips CM200) were employed for characterization of crystallinity and morphology, respectively. Zeta potential was measured by using Zeta Meter 4.0, Zeta Meter Inc. Specific surface area determinations were done by the Brunauer–Emmett–Teller (BET, Belsorp mini II, Microtrac Bel Corp) technique with the BELCAT-A instrument. Thermogravimetric analysis (TGA, L81A1750, Linseis) was employed for studying the thermal stability of products. Flame atomic absorption instrument (AAS) (Hewlett-Packard 3510) was applied for measuring the concentration of lead(II) ions in the solution.

### Batch adsorption experiments

Batch adsorption experiments were used to evaluate lead (II) removal by the PmPDA, ZnO, PmPDA@ZnO adsorbents in aquatic solution (50 mL). To specify, the effect of adsorbent amount and the lead(II) initial concentration, a series of experiments was accomplished by changing the adsorbent dosage (20–60 mg), and the initial concentration (10–90 mg/L). The pH (2–8) of the solution was adjusted by HCl and NaOH (0.1 mol/L). Each experiment was repeated three times and average results were reported. Lead(II) amount in the solution was determined by AAS. The equilibrium adsorption capacity (Q_e_) of lead(II) was calculated using the following Eq. (1).

1 $$Q_{e} = \left( {C_{i} - C_{e} /m} \right) \times V$$

where C_i_ and C_e_ are the initial and final concentration of lead(II) in the solution (mg/L) before and after adsorption, respectively, V (L) is the solution volume and m (g) is the adsorbent amount.

### Desorption and reusability

To examine of desorption and reusability of the PmPDA@ZnO adsorbent, the lead(II) adsorbed onto PmPDA@ZnO nanocomposite (50 mg) was immersed in an aqueous solution of hydrogen chloride (0.1 M) and then stirred at room temperature for 2 h. Then, the PmPDA@ZnO nanocomposite was filtered. After that, a flame atomic absorption instrument was employed for the measurement of the concentration of released lead(II) in the elution medium. The desorption percentage was measured through the following equation^33^.

2 $$\%D=\frac{A}{B}\times 100$$

where A (mg) and B (mg) are the lead(II) desorbed to the elution medium and lead(II) adsorbed on the PmPDA@ZnO nanocomposite, respectively.

### Antimicrobial activity

The antimicrobial activities of the ZnO NPs, PmPDA, PmPDA@ZnO(5%), and PmPDA@ZnO (10%) were assayed using the Kirby–Bauer disk diffusion method. 0.2 g of the powdered ZnO NPs, PmPDA, PmPDA@ZnO(5%), and PmPDA@ZnO(10%) samples were prepared separately in a tablet form and placed on the surface of inoculated agar plates. The antimicrobial activity of the samples was evaluated against *Escherichia coli* (*E*. *coli*) and *Staphylococcus aureus* (*S*. *aureus*) bacterial species. Antimicrobial activity was investigated by evaluating the inhibition zone diameter (mm) on the surface of the plate and the results were reported as Mean ± SD after three repeats.

## Results and discussion

### Characterization of adsorbent

**Figure 1:**
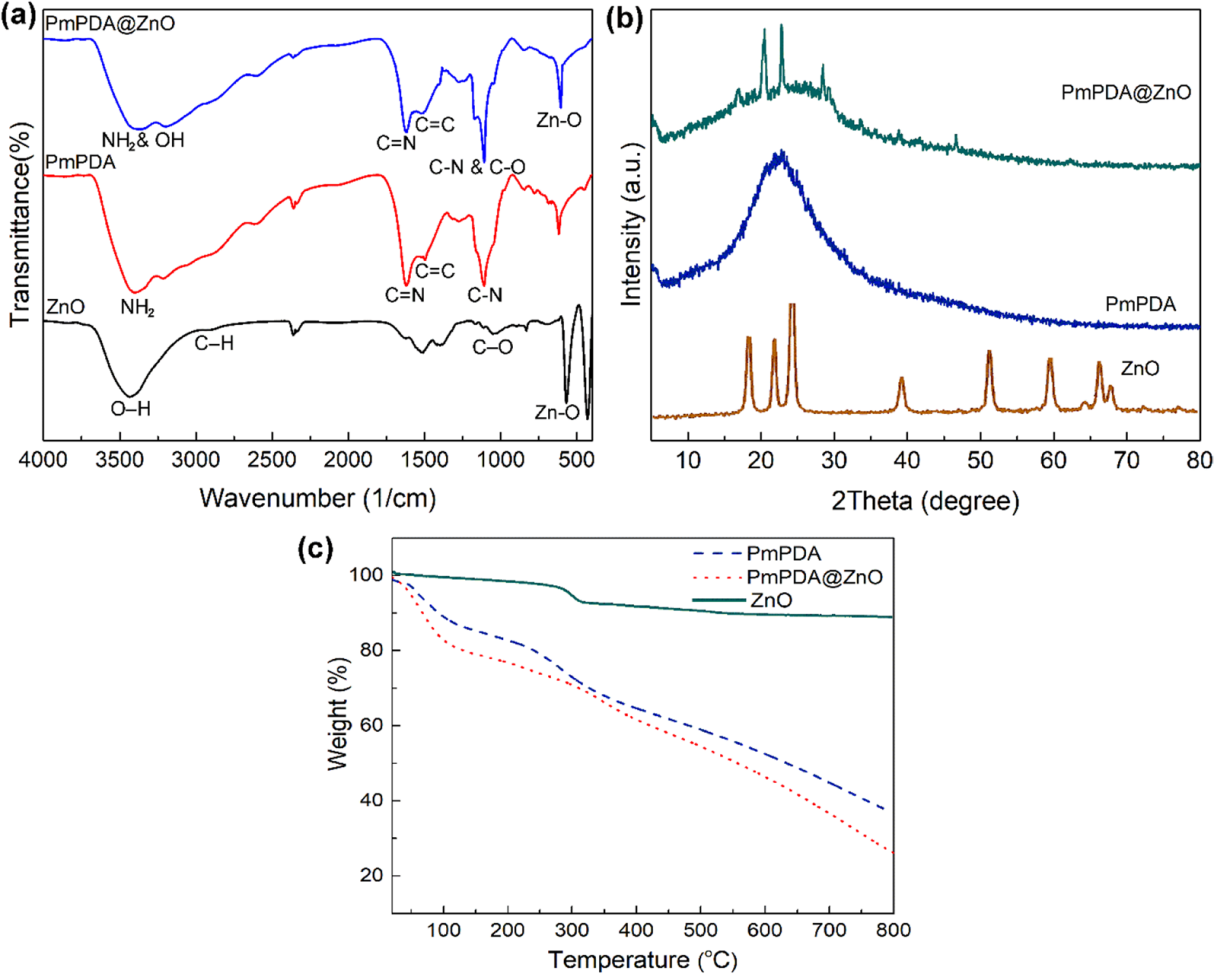
FTIR spectra (**a**), XRD patterns (**b**), and TG curves (**c**) of ZnO NPs, PmPDA, and PmPDA@ZnO.

FT-IR spectra were used to show the successful fabrication of the PmPDA@ZnO nanocomposite. Figure 1a shows the FTIR spectra of ZnO NPs, PmPDA, and PmPDA@ZnO nanocomposite. In the FTIR spectrum of ZnO NPs the absorption peaks around 522 cm^-1^, 1079 cm^-1^, and 3480 cm^-1^ are ascribed to the stretching vibrations of Zn–O, the symmetric C–O, and O–H groups, respectively^34^. The absorption peaks at 1638 cm^−1^ and 2927 cm^−1^ are related to the C = O and C–H, respectively^34^. In the FTIR spectrum of PmPDA, the characteristic absorption peaks at 3168 cm^-1^, 1625 cm^–1^, 1518 cm^–1^, and 1188 cm^−1^ are related to starching vibrations of NH_2_, C = N, C = C, and C–N, respectively^35^. In the FT-IR spectra of PmPDA@ZnO nanocomposite, the absorption peak at 3400 cm^−1^ is ascribed to O–H and NH_2_ stretching vibration modes. The absorption peaks at 1624 cm^-1^ and 1570 cm^-1^ are related to stretching vibration modes of C = N and C = C, respectively. The absorption peak at 522 cm^−1^ is related to the characteristic stretching mode of the Zn–O band. The presence of characteristic absorption peaks related to ZnO NPs and PmPDA in the FTIR spectrum of nanocomposite indicates that the PmPDA@ZnO nanocomposite was fabricated successfully.

The XRD patterns of ZnO NPs, PmPDA, and PmPDA@ZnO nanocomposite are displayed in Fig. 1b. The XRD pattern of ZnO NPs showed a crystalline nature with several diffraction peaks at 2Theta = 18.1°, 22.02°, 24.51°, 39.18°, 52.21°, 59.12°, 66.34°, and 69.13°^36^. An amorphous nature was observed at the XRD pattern of PmPDA^37^. In the XRD pattern of PmPDA@ZnO nanocomposite, significant peaks at 20.1°, 22.0°, 30.02°, 40.0°, and 47.25° correspond to the structure of ZnO NPs were observed. This shows that the presence of ZnO NPs somewhat improved the crystallinity of the nanocomposite.

The thermal stability of the ZnO NPs, PmPDA, and PmPDA@ZnO nanocomposite was studied using the TGA (Fig. 1c). A weight loss at the range 280–325 °C is observed in the TG curve of ZnO NPs which is related to the removal of trapped water molecules and the decomposition of organic components in the sample during synthesis^38^. In the TG curve of PmPDA, two weight losses are observed in the ranges of 100–280 °C and 350–800 °C. The first weight loss is related to the removal of moisture and HCl and the second weight loss is related to the removal of amine groups and the degradation of benzoid–quinoid units in the PmPDA chain^35^. Three weight losses in the ranges of 25–150 °C, 170–390 °C, and 400–800 °C are observed in the TG curve of PmPDA@ZnO nanocomposite. The first weight loss is associated with the elimination of trapped water or solvent molecules in the nanocomposite, the second weight loss is attributed to the degradation of benzoid–quinoid units in the polymer chain, and the last weight loss is related to the complete degradation of the polymer. According to the TG curves of the PmPDA and PmPDA@ZnO, it can be concluded that the thermal stability of PmPDA@ZnO is higher than the PmPDA, due to the presence of ZnO nanoparticles.

**Figure 2:**
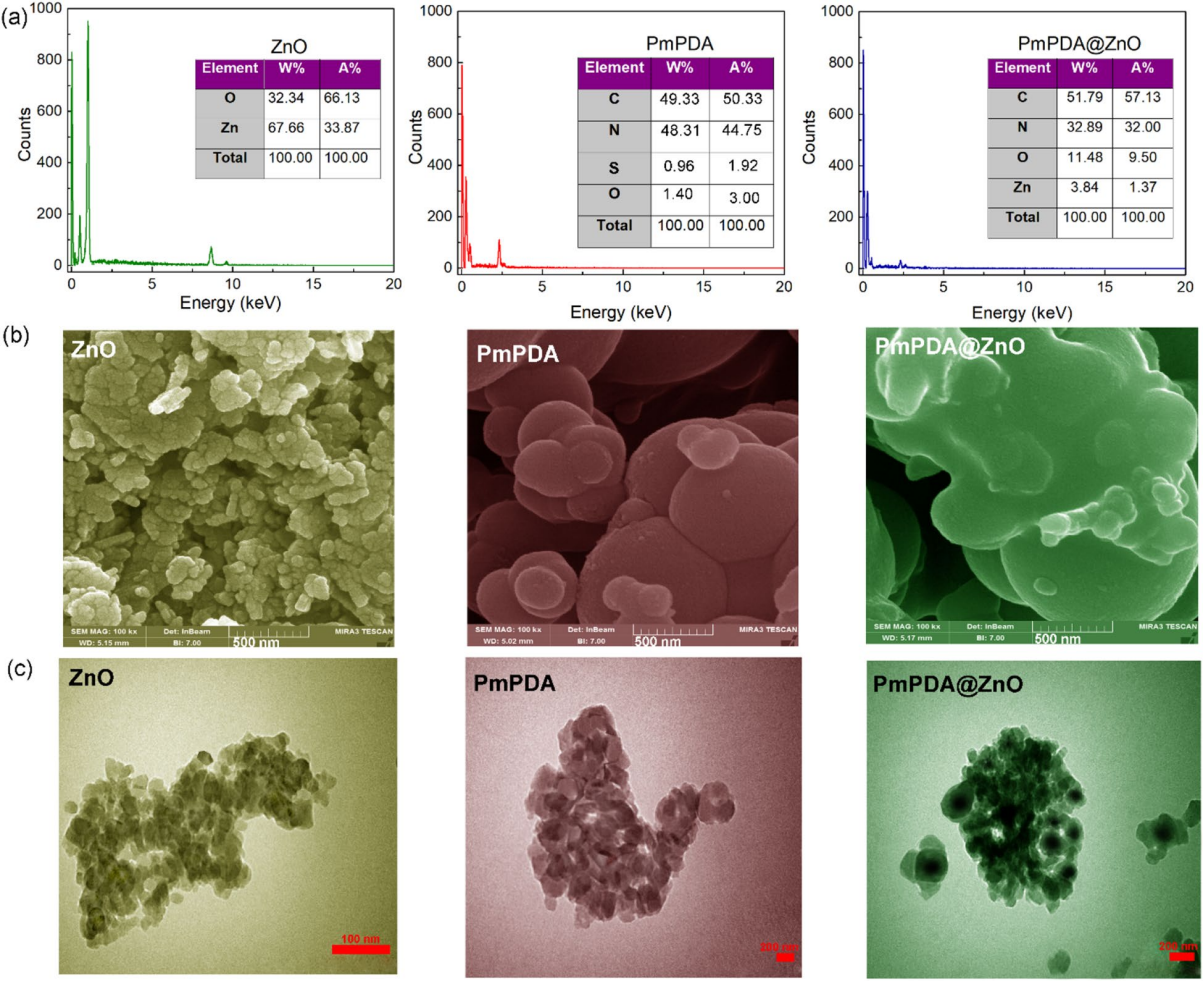
EDX spectra (**a**), FESEM (**b**) and TEM (**c**) images of ZnO, PmPDA, and PmPDA@ZnO.

EDX spectroscopy was used to determine the chemical composition of the prepared samples. Figure 2a shows the EDX spectra and tabulated data of ZnO NPs, PmPDA, and PmPDA@ZnO.

The presence of Zn and O in the EDX spectrum of the ZnO NPs and N and C elements in the EDX spectrum of PmPDA confirmed their chemical composition^39^. The presence of small amounts of O and S in the polymer was due to the presence of the APS oxidant in the synthesis of polymer^40^. The presence of the Zn element in the EDX spectrum of the PmPDA@ZnO nanocomposite showed that the PmPDA@ZnO nanocomposite was fabricated successfully.

The FESEM and TEM were employed for the morphology study of the prepared samples. Figure 2b, c show the FESEM and TEM images of the ZnO NPs, PmPDA, and PmPDA@ZnO samples at different magnification, respectively. The FESEM and TEM images of ZnO nanoparticles show a spherical-like structure with high aggregation of particles due to their high surface-to-volume ratio with a diameter between 30 and 40 nm^39^. The FESEM and TEM images of the PmPDA sample show an almost spherical structure with a diameter between 200 and 500 nm^37^. It was reported that many parameters and processes including the initiator or oxidant, the molar ratio of monomer/oxidant, pH, temperature, solvent, chemical additives, chemical oxidation process (interfacial reaction), template (hard or soft), electrochemistry, radiochemistry, and sonochemistry can be influenced the synthesis of nanostructures^41^. In this research, the synthesis of PmPDA with a diameter of 200–500 nm might be due to the pH of the medium and molar ratio of monomer/oxidant. The FESEM of the PmPDA@ZnO nanocomposite is very similar to the FESEM image of PmPDA, which shows the ZnO nanoparticles are dispersed in some areas of PmPDA matrix. According to the TEM image of PmPDA@ZnO nanocomposite, it is clear that ZnO NPs were encapsulated in PmPDA matrix (light color), creating a core–shell-like structure. Moreover, it is hard to find specific ZnO NPs and PmPDA components in the TEM images. This shows the growth of PmPDA upon ZnO NPs through in-situ polymerization.

**Figure 3:**
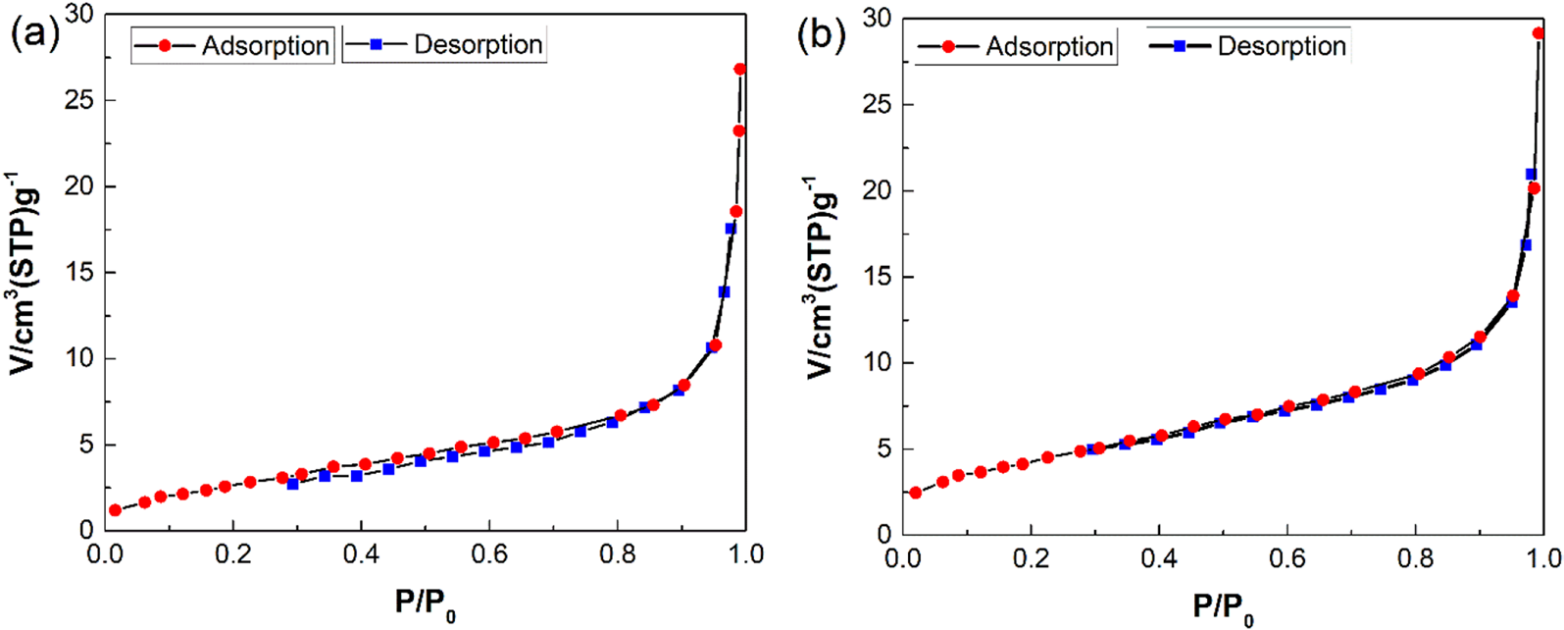
N_2_ adsorption/desorption isotherms of PmPDA (**a**) and PmPDA@ZnO (**b**).

The BET analysis was employed to define the specific surface area of prepared materials and study the impact of the ZnO NPs incorporation within the PmPDA matrix. As can be seen in Fig. 3, the specific surface area of PmPDA@ZnO was 16.019 m^2^/g which was reasonable compared to PmPDA 11.321 m^2^/g. This fact exhibition that the existence of ZnO NPs in the PmPDA matrix leads to an increase in PmPDA@ZnO surface area in comparison with PmPDA.

To study the surface charge of the PmPDA@ZnO nanocomposite as a function of pH, the zeta potential study was done potentials, and the results are displayed in Table 1. The PmPDA@ZnO nanocomposite showed negative zeta potentials in the pH range from 4 to 8. This is associated with the existence of different redox states of PmPDA@ZnO nanocomposite at different pH values. Protonation increases at pH 4 owing to the production of radical cations and the binding of protons to the amine groups, decreasing negative zeta potential. The lowering of the negative zeta potential magnitude above pH 6 because of deprotonation of the amine groups of PmPDA resulted in less oxidation and decreased negative sites.

**Table 1:** Zeta potential values of PmPDA@ZnO at room temperature.

Sample	pH	Zeta potential (mV)
PmPDA@ZnO	4	-15.09
PmPDA@ZnO	6	-28.06
PmPDA@ZnO	8	-33.10

### Lead(II) adsorption studies

#### pH

**Figure 4:**
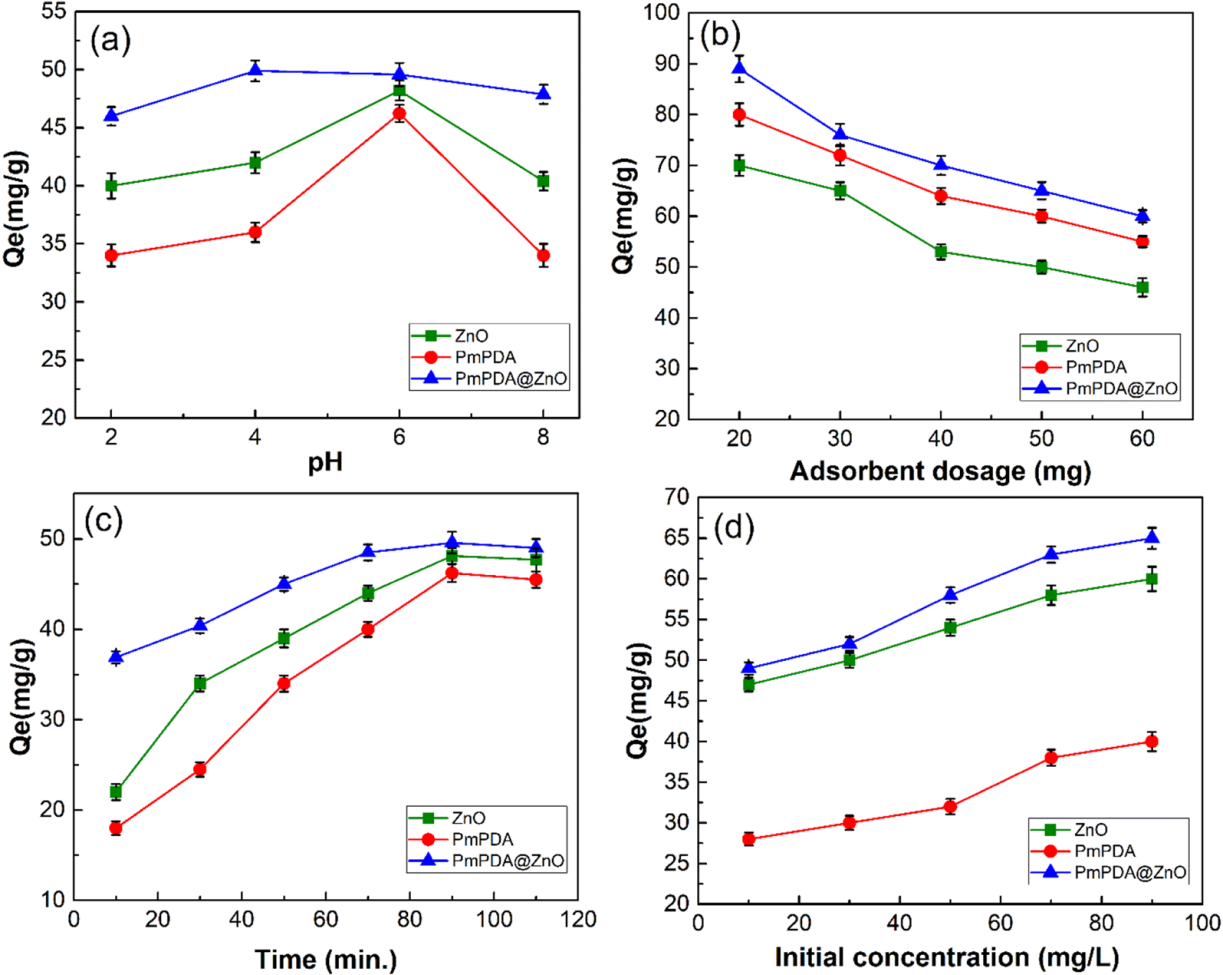
Effect of solution pH (2.0–8.0), T(298 K), adsorbent dosage (50 mg), initial concentration (50 mg/L), V (50 mL) and Time (60 min) (**a**); adsorbent dosage (20–60 mg), pH (6), T (298 K), Time (60 min), initial concentration (50 mg/L) and V(50 mL) (**b**), agitation time (10–110 min), pH (6), adsorbent dosage (60 mg), T (298 K), initial concentration (50 mg/L), and V (50 mL) (**c**), and initial concentration of lead(II) (10–90 mg/L), adsorbent dosage (60 mg), pH (6), T (298 K), and Time (90 min) (**d**).

The metal ions' adsorption is dependent on the solution pH. Figure 4a displays the pH effect on the lead (II) adsorption onto the ZnO NPs, PmPDA, and PmPDA@ZnO nanocomposite. The adsorption capacities increased with increasing pH and reached the maximum level around pH 6.0 for all the adsorbents (ZnO NPs, PmPDA, and PmPDA@ZnO). At pH > 6.0, lead(II) hydrolysis takes place, as a result, the PbCO_3_^−^ ion and Pb(OH)_2_ are formed^42^. Consequently, it would be difficult to differentiate between the adsorption and precipitation of lead(II) from solutions^42^. Thus, pH 6.0 was selected as an optimal pH for studying further adsorption experiments to avoid precipitation of Pb(OH)_2_. At pH 6.0, the removal efficiency of lead(II) was 80.50%, 81.84%, and 95.84%, for ZnO NPs, PmPDA, and PmPDA@ZnO nanocomposite, respectively. In addition, lead(II) adsorption onto PmPDA@ZnO nanocomposite was much higher compared to ZnO NPs, and PmPDA individually which can be due to the high surface-to-volume ratio and existence of amine chelating groups in PmPDA@ZnO nanocomposite.

#### Adsorbent dosage

The adsorbent capacity is a significant parameter that is determined by the adsorbent dosage for a definite initial concentration of metal ions. Figure 4b displays that the adsorption capacity increases with a decrease in the amount of ZnO NPs, PmPDA, and PmPDA@ZnO adsorbents. This could arise from the increased amount of lead(II) ions that are available to be adsorbed per unit mass of sorbent, and vice versa. The adsorption capacity of ZnO NPs, PmPDA, and PmPDA@ZnO adsorbents was 70.11, 80.01, and 90.03 mg/g with an adsorbent dosage of 20 mg, respectively. The removal efficiency of lead(II) reach 95% (PmPDA), 97% (ZnO), and 99% (PmPDA@ZnO) when the adsorbent dosage is 60 mg. Consequently, 60 mg was chosen as the optimal dosage of adsorbents for the subsequent experiments.

#### Agitation time

The adsorption efficiency of lead (II) onto adsorbents is related to the adsorption time. Figure 4c displays the influence of contact time on the adsorption capacity of ZnO NPs, PmPDA, and PmPDA@ZnO adsorbents. With increasing contact time, the adsorption efficiency increased considerably up to 90 min and then remained constant. According to the results, the adsorption capacity reached the equilibrium values of 46.83, 45.86, and 49.59 mg/g for ZnO NPs, PmPDA, and PmPDA@ZnO, respectively. Adsorption was fast initially because the concentration of lead(II) and also the number of free adsorptive sites are high. At a later stage, adsorption slowed down and reached an equilibrium level because of a decrease in lead(II) concentration and also exhaustion of free adsorptive sites. Thus, 90 min was selected as the optimum agitation time for the subsequent experiments.

#### Initial lead(II) concentration

Figure 4d displays the effect of the initial concentration of lead(II) on the adsorption capacity of ZnO NPs, PmPDA, and PmPDA@ZnO adsorbents. As the initial concentration of lead (II) increases, the adsorption capacity first increased linearly and finally reached the maximum value. The adsorption capacity of the adsorbents will not increase above the 90 mg lead(II) concentration. This might be that at the higher initial concentration of lead(II) the total existing adsorption sites are confined, therefore resulting in a decrease of adsorption capacity.

### Adsorption isotherm

**Figure 5:**
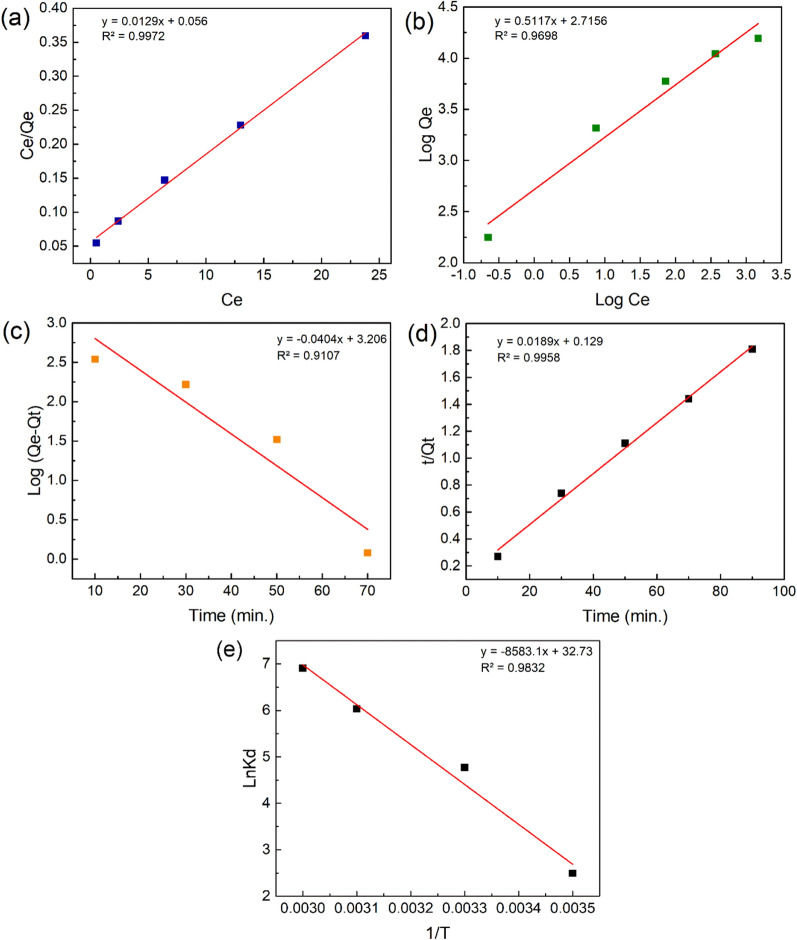
The Langmuir (**a**) and Freundlich (**b**) isotherm models (lead (II) initial concentration: 10–90 mg/L, adsorbent dosage: 60 mg, pH: 6, T: 298 K, and agition time: 90 min); Pseudo-first-order (**c**) and pseudo-second-order (**d**) kinetic models (lead (II) initial concentration: 90 mg/L, adsorbent dosage: 60 mg, pH: 6, T: 298 K, and agition time: 30–90 min); and thermodynamic model (**e**) (Temperature: 288–328 K; adsorbent dosage: 60 mg; pH: 6, lead (II) initial concentration: 90 mg/L; agitation time: 90 min).

According to batch adsorption studies, we employed only PmPDA@ZnO nanocomposite for studying isotherm, kinetics, and thermodynamics. Adsorption isotherms were employed to determine the equilibrium relationship between lead (II) ions and PmPDA@ZnO nanocomposite at a constant temperature. Langmuir and Freundlich isotherm models were employed and the information regarding these models is presented in Fig. 5a, b and Table 2. The Langmuir isotherm assumes that sorption is accomplished at definite homogeneous places inside the sorbent and the adsorption process is a monolayer. On the other side, the Freundlich isotherm assumes that sorption takes place on heterogeneous surfaces. The Langmuir and Freundlich isotherm equations are expressed by following Eqs. (3), and (4) respectively.

3 $$\frac{{C}_{e}}{{Q}_{e}}=\frac{1}{{K}_{L}{Q}_{max}}+\frac{1}{{Q}_{max}}{C}_{e}$$

4 $$Log{Q}_{e}=\mathrm{log}{K}_{f}+\frac{1}{n}\mathrm{log}{C}_{e}$$

where, Ce is the equilibrium concentration of metal ions (mg/L); Q_e_ and Q_max_ are the equilibrium and maximum adsorption capacity (mg/g), respectively; K_L_ (L/mg) and K_F_ (L/mg) are the Langmuir and Freundlich constants calculated from the plot between Ce/Q_e_ and Ce, and between log Q_e_ and log C_e_, respectively. Table 2 showed that the correlation coefficient (R^2^) value of the Langmuir model was higher than the Freundlich model. Thus, lead (II) adsorption onto PmPDA@ZnO was more fitted to the Langmuir model. Moreover, the maximum adsorption capacity was calculated to be 77.51 mg/g from the Langmuir model.

**Table 2:** Isotherm, kinetic, and thermodynamic parameters for adsorption of lead(II) onto PmPDA@ZnO nanocomposite.

Model	Parameters		
Isotherm	Langmuir	Q_m_(mg/g)	77.51
		K_L_(L/mg)	0.23
		R^2^	0.99
	Freundlich	K_F_(mg/g)	15.11
		n	1.95
		R^2^	0.96
Kinetic	Pseudo-first-order	Q_e_(mg/g)	24.68
		k_1_(min^-1^)	-0.04
		R^2^	0.91
	Pseudo-second-order	Q_e_(mg/g)	49.50
		k_2_(min^-1^)	0.27
		R^2^	0.99
Thermodynamic		ΔG°(kJ/mol)	-5.96 (288)
			-11.82 (298)
			-15.94 (318)
			-18.84 (328)
		ΔH°(kJ/mol)	71.35
		ΔS°(J/mol K)	0.272

### Adsorption kinetics

The kinetic study is very significant in batch experiments to find the optimum interaction time of metal ions with sorbents. Consequently, to find the adsorption mechanism, the pseudo-first-order (Eq. 5) and pseudo-second-order (Eq. 6) models were employed and the information concerning the models is shown in Fig. 5c, d and Table 2.

5 $$Log\left({Q}_{e}-{Q}_{t}\right)=Log{Q}_{e}-\frac{{k}_{1}}{2.303}t$$

6 $$\frac{t}{{Q}_{t}}=\frac{1}{{k}_{2}{Q}_{e}^{2}}+\frac{1}{{Q}_{e}}t$$

where Q_t_ (mg/g), and Q_e_ (mg/g) are the adsorption capacity (or amount of lead(II) adsorbed onto sorbent) at time and equilibrium, respectively. K_1_ (1/min) and K_2_ (g/mg.min) are the rate constants of the pseudo-first-order and pseudo-second-order, respectively. The R^2^ value for the pseudo-second-order kinetic model was better and closer to one than that of the pseudo-first-order kinetic model which determined that the pseudo-second-order kinetic model was more suitable for fitting the experimental data.

### Adsorption thermodynamic

Temperature is an important factor in the adsorption process and could be altered the adsorption capacity of the adsorbent. The temperature effect on the lead(II) adsorption by the PmPDA@ZnO nanocomposite was investigated at 288–328 K. The temperature dependence on the sorption process is associated with numerous thermodynamic parameters e.g. Δ*G*°, Δ*H*°, and Δ*S*°. The Δ*H*°, Δ*G*°, and Δ*S*° values were measured from the Eqs. (7), (8), and (9) and are shown in Table 2.

7 $$K_{d} = Q_{e} /C_{e}$$

8 $$\Delta G^{ \circ } = - RTLnK_{d}$$

9 $$LnK_{d} = \left( {\Delta S^{ \circ } /R} \right) - \left( {\Delta H^{ \circ } /RT} \right)$$

where *K*_*d*_, R, and T are the distribution coefficient (mL/g), the universal gas constant (8.314 J/mol K), and absolute temperature (K), respectively. Δ*H*° (J/mol) and Δ*S*° (J/mol K) are enthalpy and entropy changes, respectively. The Δ*H*° and Δ*S*° values were measured from the slope and intercept of the linear plot of Ln *K*_*d*_ vs. 1/T as revealed in Fig. 5e. The increase in the Δ*G*° values with increasing the temperature (Table 2) revealed that the lead(II) adsorption onto PmPDA@ZnO nanocomposite was more favorable at lower temperatures. The positive Δ*H*° value (71.35 kJ/mol) proposed the endothermic nature of lead (II) adsorption onto PmPDA@ZnO. It is well known that the Δ*H*° values in physical and chemical adsorptions lie between 2.1- 20.9 kJ/mol and 20.9–418.4 kJ/mol, respectively^43–45^. Thus, the adsorption of lead (II) onto PmPDA@ZnO was chemisorption. Moreover, the value of Δ*S*° revealed the possibility of increased changes at the solid/liquid interface during the adsorption of lead (II) onto PmPDA@ZnO.

### Comparison of current results with other literature

For comparison of current results with other literature, the maximum adsorption capacity (Q_m_) of several adsorbents reported in the literature is listed in Table 3. Data in Table 3 expose that the adsorption capacity of the PmPDA@ZnO is much higher than that of the reported adsorbents, indicating that the PmPDA@ZnO nanocomposite can be effectively applied for the removal of lead(II) ions from aqueous solutions. The better Q_m_ of PmPDA@ZnO nanocomposite than other adsorbents might be owing to the existence of amine groups and acceptable specific surface area at the PmPDA@ZnO nanocomposite which can bind with lead(II) ions through physicochemical interactions.

**Table 3:** Comparison of the maximum adsorption capacities of lead(II) by various adsorbents.

Adsorbents	Q (mg/g)	References
PmPDA@ZnO	77.51	Current study
Polyaniline-*grafted-*chitosan	16.07	^46^
Polyaniline@Sb_2_O_3_	21.05	^47^
Polyaniline @Attapulgite	15.42	^48^
Polythiophen@Sb_2_O_3_	18.94	^49^
Anthranilic acid/4-nitroaniline@formaldehyde resin	7.64	^50^
Reduced graphene oxide@Fe_3_O_4_	30.68	^51^
Modified Preyssler@chitosan@Fe_3_O_4_	25.9	^52^
Chitosan-*g*-poly(acrylamide)@Cu nanocomposite	38.93	^53^
Zeolite/ZnO nanocomposite	47.6	^54^
TiO_2_/Graphene oxide nanocomposite	65.6	^55^
ZnO/talc nanocomposite	48.3	^27^

### Effect of coexisting cations

**Figure 6:**
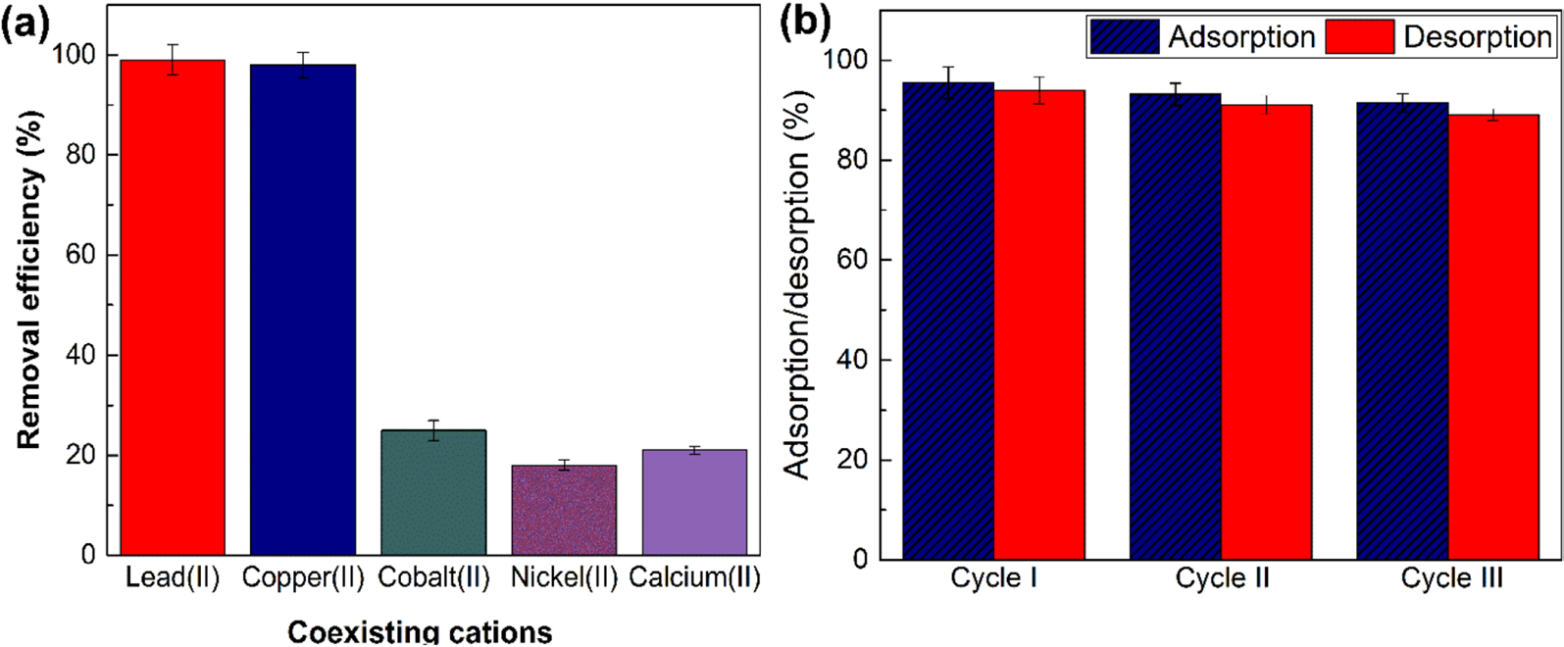
Effect of coexisting ions on lead(II) removal (**a**). pH 6, adsorbent dose: 60 mg/L, initial cation concentration: 50 mg/L, contact time: 90 min, T: 298 K. Reusability of the PmPDA@ZnO, adsorption/desorption percentages of lead(II) during three cycles (**b**).

Adsorption of various metal cations e.g. cobalt (II), nickel (II), calcium (II), and copper (II) as coexisting cations onto PmPDA@ZnO nanocomposite was evaluated. A solution containing a mixture of lead(II) with the aforementioned cations was prepared at pH 6. The cations concentration was retained at 50 mg/L (Fig. 6a). Results were repeated three times and average results were reported. Remarkably, the existence of these coexisting cations has no significant influence on lead (II) removal efficiency. Moreover, cobalt (II), nickel (II), calcium (II), and copper (II) were able to adsorb onto the PmPDA@ZnO nanocomposite. It was reported that the competitive adsorption ability differs from one metal ion to another and is associated with numerous factors, e.g. molecular mass, ion charges, hydrated ionic radius, and hydration energy of the metals^56^. Thus, the coexisting cations did not compete with lead (II) ions for the active sites on the PmPDA@ZnO nanocomposite, proposing multi-surface adsorption^57^.

### Recovery and reusability

The adsorbent retrievability and reusability are important in terms of environmental protecting and saving cost, time, and energy because adsorbents with these properties excellently reduce the problems of used adsorbent disposal and the new adsorbent production process^58^. Adsorption and desorption experimental tests were performed for three consecutive cycles to estimate the recoverability and regeneration of the PmPDA@ZnO nanocomposite. The desorption of lead (II) ions from the nanocomposite adsorbent were done out by immersing and stirring the nanocomposite adsorbent in HCl solution (0.1 M) at room temperature for 2 h. Lead (II) ions were released into the solution and the adsorbent was filtered and washed several times with distilled water and dried for consequent adsorption/desorption experiments. As is observed in Fig. 6b, the adsorption percentage decreased from 96.34% to 91.25% and the desorption percentage decreased from 93.14% to 88.89% after the three cycles. These results showed that the PmPDA@ZnO nanocomposite retains the ability to lead (II) removal after three consecutive adsorption–desorption cycles, without significant reduction in the adsorption performance.

### Suggested mechanisms of adsorption

**Figure 7:**
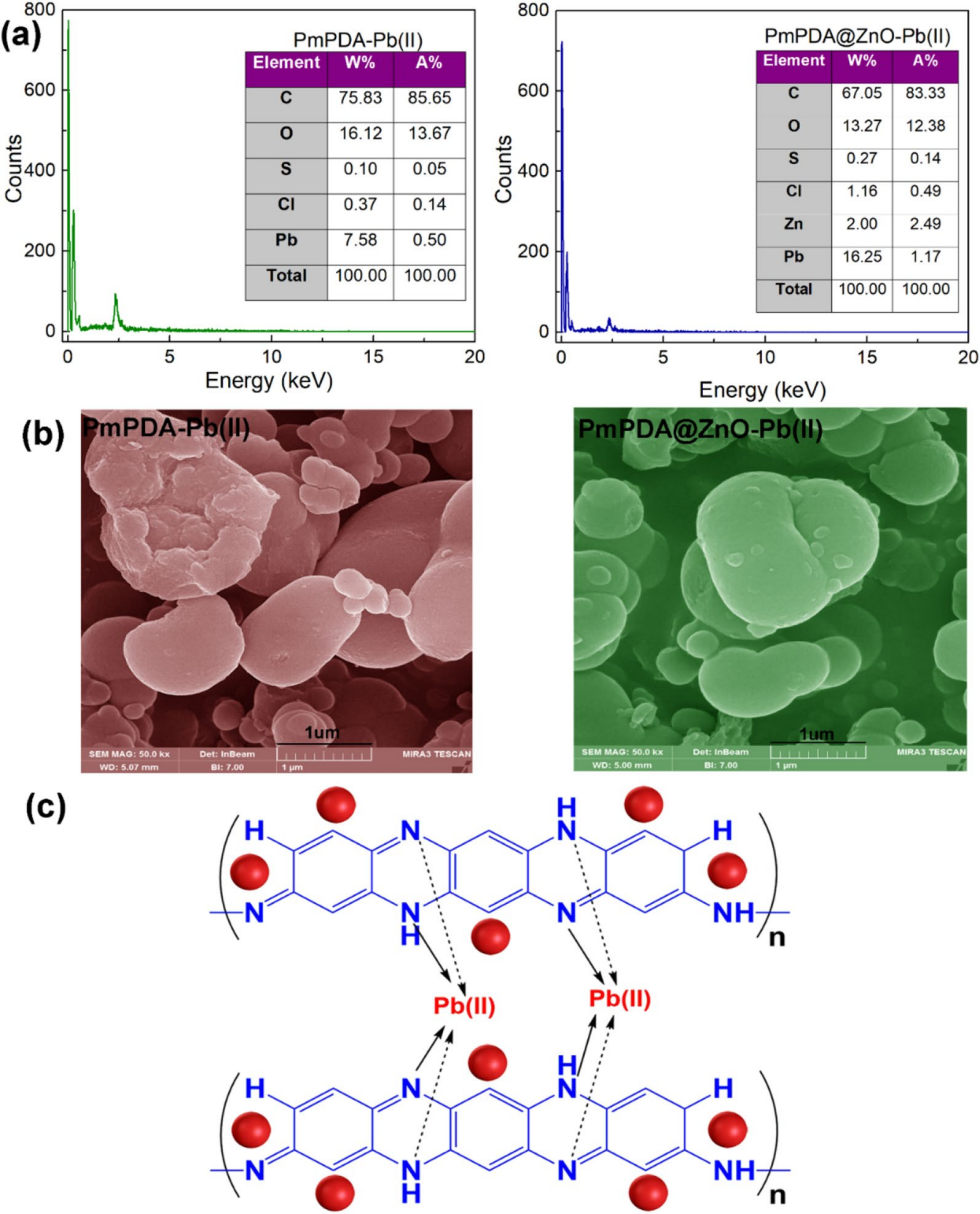
EDX spectra (**a**) and FESEM images (**b**) of the PmPDA and PmPDA@ZnO nanocomposite after the sorption of lead(II) ions. A mechanism for the adsorption of lead(II) ions onto PmPDA@ZnO nanocomposite (**c**).

FESEM and EDX were employed for the confirmation of lead(II) adsorption onto PmPDA and PmPDA@ZnO nanocomposite (Fig. 7a, b). As can be seen, the FESEM images of PmPDA and PmPDA@ZnO adsorbents have changed after complexing with lead(II) ions. In addition, the presence of lead elements in the EDX spectra of PmPDA and PmPDA@ZnO nanocomposite indicates the adsorption of lead(II) ions on adsorbents. On the other hand, the adsorption of lead (II) ions onto PmPDA@ZnO nanocomposite was higher than the PmPDA, which indicated the higher performance of PmPDA@ZnO nanocomposite than the PmPDA. This could be due to the presence of ZnO nanoparticles with a high surface-to-volume ratio.

On the other side, the functional groups of PmPDA@ZnO nanocomposite play an important role in the adsorption process of lead(II) ions. At pH 6 a considerably high electrostatic attraction occurs between the amine groups of the adsorbents (PmPDA and PmPDA@ZnO nanocomposite) and the lead(II) ions. As the pH of the solution decreases pH < 6, the number of positively charged sites increases, and the number of negatively charged sites decreases. A positively charged surface site on the PmPDA and PmPDA@ZnO adsorbents does not favor the adsorption of lead(II) ions, owing to the electrostatic repulsion. Furthermore, at low pH (2–4) values, excess hydrogen ions compete with the lead(II) ions for the adsorption site resulting in lower adsorption of lead(II) (Fig. 7c).

### Antibacterial activity

**Figure 8:**
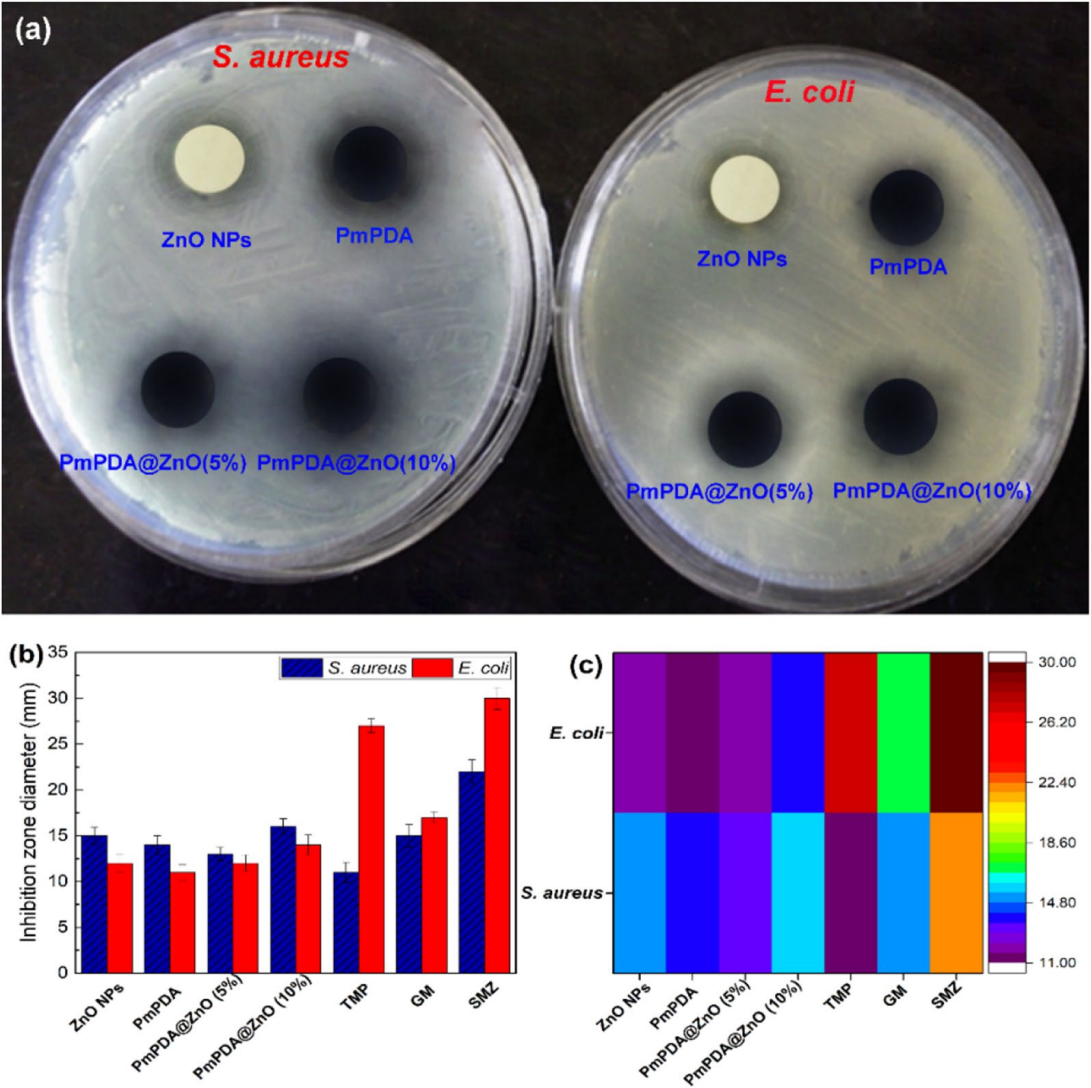
Evaluating antimicrobial activity by agar disk diffusion (**a**), histogram (**b**), and heat map (**c**) of ZnO NPs, PmPDA, PmPDA@ZnO (5%), and PmPDA@ZnO (10%) against bacterial pathogens. Trimethoprim (TMP), sulfamethoxazole (SMZ), and gentamicin (GM).

The antimicrobial activity of ZnO NPs, PmPDA, PmPDA@ZnO(5%), and PmPDA@ZnO(10%) against two bacterial species, Gram-negative (*Escherichia coli*) and Gram-positive bacteria (*Staphylococcus aureus*) was studied (Fig. 8a). Antimicrobial activity was evaluated by measuring the inhibition zone diameter after 24 h of incubation and compared with trimethoprim (TMP), sulfamethoxazole (SMZ), and gentamicin (GM) antibiotics (Fig. 8b, c). Results demonstrate the difference of the diameter zone and the activity index in the ZnO NPs, PmPDA, PmPDA@ZnO (5%), and PmPDA@ZnO (10%). The results proved that increasing the ratio of ZnO NPs in the nanocomposite increases the antibacterial activity against the tested bacterial species but the best inhibition effect was against *S. aureus*. This synergistic effect of antimicrobial activity can be due to the presence of ZnO NPs as well as PmpDA in the nanocomposite^59,60^.

## Conclusions

In this research, the PmPDA@ZnO nanocomposite with core–shell-like structure was prepared by in-situ polymerization and employed as an effective sorbent for the toxic lead(II) ions removal from the aqueous solutions. The BET analysis showed that the specific surface area of PmPDA@ZnO nanocomposite was 16.019 m^2^/g compared to PmPDA with 11.321 m^2^/g. Moreover, FTIR, EDX, TEM, and TGA analyses confirmed the presence of ZnO NPs in the PmPDA matrix. The fabricated PmPDA@ZnO nanocomposite displayed a maximum adsorption capacity of 77.51 mg/g at pH 6, time 90 min and 298 K. The adsorption isotherms and kinetics showed a better fit with the Langmuir model and pseudo-second-order kinetics, respectively. The adsorption of lead (II) onto PmPDA@ZnO nanocomposite was chemisorption. Δ*G*° values showed that the lead(II) adsorption onto PmPDA@ZnO nanocomposite was more favorable at lower temperatures. Adsorption–desorption results displayed that the PmPDA@ZnO nanocomposite retains the ability to lead (II) removal after three consecutive adsorption–desorption cycles, without significant reduction in the adsorption performance. The antimicrobial results proved that increasing the ratio of ZnO NPs increases the antimicrobial activity against the bacterial species but the best inhibition effect was against *S. aureus*. In summary, the simple preparation, relatively high adsorption capacity, and good antimicrobial activity showed that the PmPDA@ZnO nanocomposite can be successfully employed for water decontamination.
